# Impact of time‐restricted feeding on kidney injury in male rats with experimental metabolic syndrome

**DOI:** 10.1113/EP091145

**Published:** 2023-04-19

**Authors:** Rajendran Harishkumar, Eva M. Baranda‐Alonso, William P. Martin, Neil G. Docherty

**Affiliations:** ^1^ Diabetes Complications Research Centre, School of Medicine Conway Institute, University College Dublin Dublin Ireland; ^2^ Institute Biomedical Research of Salamanca (IBSAL) Paseo de San Vicente Salamanca Spain; ^3^ Department of Physiology and Pharmacology University of Salamanca Salamanca Spain

**Keywords:** albuminuria, high‐fat diet, kidney injury, metabolic syndrome, time‐restricted feeding

## Abstract

Disruptions to circadian rhythm may be implicated in the pathogenesis of metabolic syndrome (Met‐S). For example, eating during an extended period of the day may negatively impact the circadian rhythms governing metabolic control, contributing, therefore, to Met‐S and associated end‐organ damage. Accordingly, time‐restricted eating (TRE)/feeding (TRF) is gaining popularity as a dietary intervention for the treatment and prevention of Met‐S. To date, no studies have specifically examined the impact of TRE/TRF on the renal consequences of Met‐S. The proposed study seeks to use a model of experimental Met‐S‐associated kidney disease to address this knowledge gap, disambiguating therein the effects of calorie restriction from the timing of food intake. Spontaneously hypertensive rats will consume a high‐fat diet (HFD) for 8 weeks and then be allocated by stratified randomisation according to albuminuria to one of three groups. Rats will have free 24‐h access to HFD (Group A), access to HFD during the scheduled hours of darkness (Group B) or access to HFD provided in the form of two rations, one provided during the light phase and one provided during the dark phase, equivalent overall in quantity to that consumed by rats in Group B (Group C). The primary outcome measure will be a change in albuminuria. Changes in food intake, body weight, blood pressure, glucose tolerance, fasting plasma insulin, urinary excretion of C‐peptide and renal injury biomarkers, liver and kidney histopathology and inflammation, and fibrosis‐related renal gene expression will be assessed as secondary outcomes.

## 1 INTRODUCTION

Metabolic syndrome (Met‐S) is a clinical diagnosis defined by a cluster of cardiovascular risk factors including insulin resistance, impaired glucose metabolism, hypertension, abdominal obesity and dyslipidaemia (Scuteri et al., 2015). Met‐S is highly prevalent in Western societies, affecting approximately a quarter of the general European population (Scuteri et al., 2015). The International Diabetes Federation estimates that globally up to 25% of the adult population is living with overweight and obesity in association with this cluster of risk factors (Ogurtsova et al., 2017). A large body of evidence drawn from meta‐analyses conducted over the last 5 years shows a strong association between Met‐S and chronic kidney disease (CKD) risk, moreover demonstrating that risk accrues as a function of the number and severity of each component of Met‐S (Alizadeh et al., 2018; Thomas et al., 2011; Wu et al., 2022, 2021). A cause‐and‐effect relationship in the association is borne out by the well‐recognised pathogenic roles of Met‐S components as systemic drivers of renal injury (Raimundo & Lopes, 2011).

Evidence indicates that defective integration of metabolism, such as that seen in Met‐S, can arise from disrupted circadian rhythm, including extensions in the proportion of the day during which food is consumed (Hawley et al., 2020). An association between insulin resistance (a key feature of Met‐S) and urinary albumin excretion (a key indicator of CKD) is reported in numerous studies (Alqallaf et al., 2022), including registry data and acute challenge‐based protocols (Ahlqvist et al., 2018; Tsuda et al., 2018), suggesting that interventions that address fundamental features of Met‐S could have a knock‐on benefit regarding the progression of CKD. Retrospective analysis of outcomes in recipients of bariatric surgery with type 2 diabetes and CKD supports this contention (Raverdy et al., 2022).

Addressing the timing, quality and quantity of food intake has emerged as a dietary intervention in patients with Met‐S. Regularised eating patterns are known to entrain peripheral molecular clocks for coordinated physiological function (Chaix et al., 2019). Time‐restricted eating (TRE) – limiting daily food intake to a 10‐h window – improves metabolic and cardiovascular parameters in individuals with Met‐S; reducing fasting insulin by 20% and peripheral insulin resistance by 30% (Wilkinson et al., 2020). There is an increasing appreciation that a molecular clock‐based mechanism underpins circadian rhythm in kidney function, integrating it with whole‐body physiology (Firsov & Bonny, 2018). Hence, the present protocol aims to determine whether a chrononutritional approach to Met‐S may be beneficial vis‐à‐vis associated renal disease. Current scientific debate centres on whether the advantages of TRE in humans depend on timing of food intake per se or arise merely as a result of reductions in daily calorie intake (Liu et al., 2022; Xie et al., 2022). Deciphering the relative roles in human studies is difficult due to factors such as inter‐individual heterogeneity in protocol compliance and differences in dietary composition.

No study to date in rodents or humans has sought to focus on examining the impact of time‐restricted feeding (TRF)/TRE on the renal consequences of Met‐S. Hence, the proposed study seeks to use the high‐fat diet (HFD) fed spontaneously hypertensive rat (SHR) model of experimental Met‐S‐associated kidney disease (Yanagihara et al., 2016) to address this knowledge gap, in a fashion that will disambiguate the effects of calorie restriction from the timing of food intake.

## 2 METHODS

### 2.1 Ethical approval

Ethical approval for the study was granted by the University College Dublin Animal Research Ethics Subcommittee (AREC‐22‐23‐Docherty). Regulatory approval of the project protocol was obtained from the Health Products Regulatory Agency of Ireland (ref. AE18982/P222). A time‐stamped preliminary research protocol for the study has been registered and made public at both Open Science Forum https://osf.io/g29ye/ and https://preclinicaltrials.eu/ (ID PCTE0000347).

### 2.2 Study design

The study design has been planned to conform with the requirements of the ARRIVE Guidelines 2.0 (Percie du Sert et al., 2020). The study will be conducted during the second quarter of 2023, with the final analysis original research manuscript submission anticipated to occur during quarter 3 of 2023.

#### 2.2.1 Experimental model (Figure 1)

Eighteen male SHRs (strain code 007, 5–6 weeks of age (Charles River Ltd, Harlow, UK) will be housed under standard vivarium conditions (room temperature: 22 ± 3°C with 12 h light–dark cycle) with ad libitum access to a standard pelleted diet and drinking water during the first 2 weeks of acclimatisation at the UCD Biomedical Facility.

**FIGURE 1:**
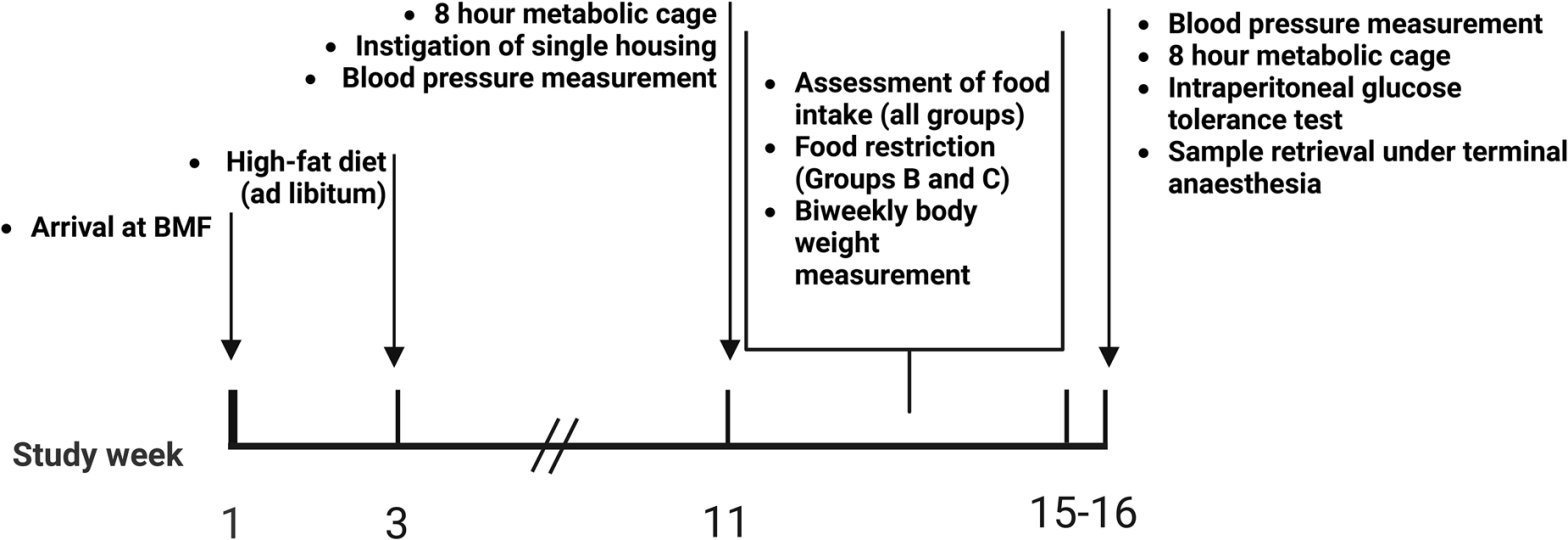
Study design diagram. BMF, biomedical facility.

After 2 weeks, animals will start to consume a HFD (260‐HF, 60% of energy from butter fat,‐SAFE Custom Diets, Augy, France) for 8 weeks. After 8 weeks, animals will be housed in metabolic cages for an 8‐h urine collection for the determination of albuminuria. Animals will then be randomised using stratified allocation into three groups according to tertiles of albuminuria as subsequently described. At this point, animals will be singly housed. After 2 days of monitoring, the average food intake across individually housed animals from all groups will be determined. Thereafter, rats in Group A (*n* = 6) will continue to have free 24‐h access to HFD. Animals in the TRF group (Group B, *n* = 6) and the equicaloric intake group (Group C, *n* = 6) will have controlled access to food and their total ration will be based on the average food intake measured during the dark phase. Rats in Group B will have access to HFD for 12 h daily during the scheduled hours of darkness (19.00–07.00 h). Rats in Group C will have access to HFD provided in the form of two rations: one provided at the light phase, and one provided at the dark phase. The quantity provided at each ration to rats in Group C will be adjusted to ensure that total intake over 24 h will be equivalent to the total daily quantity consumed during the dark phase by rats in Group B. All animals will continue to be individually housed for 4 weeks. Daily food intake and body weight (twice weekly) will be recorded over 4 weeks, and blood pressure will be measured at baseline and 4 weeks after treatment group allocation. During the fifth week after the establishment of the experimental groups, over three consecutive days, six animals per day (two from each group) will undergo an intraperitoneal (i.p.) glucose tolerance test following a 6‐h fasting period. Immediately after this, an 8‐h dark phase urinary collection will be made.

Rats will be killed in groups of six under terminal anaesthesia using inhaled anaesthetic and an analgesic. During the terminal procedure, the abdomen of the rats will be opened and samples of blood, kidneys, liver, fat tissue and heart will be collected for further analysis.

#### 2.2.2 Stratified allocation

Rats will be randomized into Groups A, B or C after 8 weeks of HFD. To prevent allocation bias, sealed and anonymised allocation envelopes will be prepared for rats across each tertile of baseline urinary albumin excretion. Allocation envelopes will be shuffled and the rats within each tertile will be assigned in pairs to Group A, B or C. Researchers will not be blinded to group allocation during the protocol.

### 2.3 Procedures and analyses

#### 2.3.1 Metabolic cage studies

Eight‐hour urine collection will be carried out between the hours of 23.00 and 07.00 at baseline after 8 weeks of ad libitum HFD consumption and again at study close after 4–5 weeks of group‐specific dietary regulation. A food reward will be provided upon entry to the cage. Urine output will be collected into clean receptacles and decanted into sterile 50‐ml plastic tubes. Urine volume will be measured, then samples will be centrifuged at 4000 *g* for 5 min to pellet any sediment present in the urine. The collected supernatant will be aliquoted and stored at −80°C. Animals will undergo two separate 1‐h sessions of acclimatisation (with food reward) to metabolic cages ahead of the baseline urine collection.

#### 2.3.2 Blood pressure measurement

Blood pressure will be measured between 08.00 and 10.00 h and again between 17.00 and 19.00 h at baseline and again at the same time, 4 weeks after allocation to experimental groups. The measurements will be performed using the CODA^®^ Non‐invasive blood pressure (BP) system (Kent Scientific, Torrington, CT, USA). The CODA system utilises a clinically validated volume–pressure recording (VPR) technology to detect the changes in tail volume that correspond to the BP of animals using a VPR cuff and an occlusion tail cuff (Hoffman et al., 2018; Suresh et al., 2019). Animals will be gently guided into a large‐size restrainer tube supplied by Kent Scientific. The nose cone and rear cap are securely fastened onto the tube, with the animal's nose protruding through a hole in the nose cone, and the tail through a hatch at the rear cap. The restrainer tube with a protruding tail will be placed on a paper towel on a warming platform pre‐warmed for 15 min beforehand at 37°C. Then the occlusion cuff will be slid onto and over the animal's tail. The tail temperature will be monitored using an infrared thermometer; a tail temperature of at least 32°C is required to ensure sufficient tail blood volume for volumetric detection of BP. As part of the session, two acclimatisation cycles and 10 cycles of recording will be initiated. Animals will be acclimatised to the restrainer apparatus for 5, 10 and then 20 min during 2 weeks before the first blood pressure measurement and these sessions will be paired with a food reward.

#### 2.3.3 Single housing

All animals will be individually housed during the period of group allocation. Animals will be acclimatised to individual cage occupancy twice for 2 and 6 h, respectively, over the 2 weeks before single housing. During the period of single housing, as a welfare refinement, animals will be regrouped with animals from their home cage for 2 h twice weekly in a rat playpen. Moreover, individually housed animals will be provided with a novel object (toys) changed twice a week.

#### 2.3.4 Intraperitoneal glucose tolerance test (4 weeks after allocation to experimental groups)

After 6 h of fasting from 13.00 to 19.00 h, an i.p. glucose tolerance test will be performed over 2 h between 19.00 and 21.00 h as previously described (Nair et al., 2020). The animal will be gently lifted and restrained by the thorax and hind limbs. i.p. glucose (1 g/kg dose delivered in 1 ml bolus) will be administered in the lower right quadrant of the abdomen to avoid the typical lower left quadrant location of caecum in rats. The needle (27G) will be inserted bevel‐side up at a 30–40° angle when entering the abdominal cavity until it penetrates about 3–4 mm. The glucose solution will be injected slowly, and the needle will be carefully withdrawn. The animal will be returned to the cage and monitored for 5 min.

Tail vein blood sampling (tail‐prick) will be performed at 0, 15, 30, 60, 90 and 120 min after i.p. glucose administration, and blood glucose levels will be measured with a glucometer (Glucomen‐areo Menarini Diagnostics, Florence, Italy). Fasting insulin will be assessed in the baseline time‐point 0 blood samples using an ultra‐sensitive rat insulin enzyme‐linked immunosorbent assay (ELISA) kit (90060 Crystal Chem (Europe), Zaandam, Netherlands). EMLA cream (Lloyd's Pharmacy, Dublin, Ireland) will be applied to the rat's tail 30 min before piercing the tail vein to provide for local anaesthesia. Vasodilatation will be achieved by warming the tail for 1 min using a glove filled with warm water. The collection point will be cleaned gently with an antiseptic solution and then the animal will be lightly restrained using a towel. The skin and the underlying blood vessel will be gently punctured with a 27G hypodermic needle.

#### 2.3.5 Laparotomy and sample retrieval

At close study, animals will be killed under terminal anaesthesia with isoflurane. One hour before anaesthesia, animals will receive a subcutaneous dose of buprenorphine (0.05 mg/kg). Once the animal is under deep anaesthesia, it will be placed supine on the operating table and a mid‐line laparotomy will be performed. The intestines will be reflected and the aorta will be exposed below the origin of the renal arteries. The wall of the aorta will be firmly held with fine forceps, with the claws approximately 0.5–1.0 cm from its distal bifurcation. Then a bent needle will be inserted into the lumen of the aorta and 5 ml of trunk blood will be collected. After this, in very rapid succession, the inferior vena cava will be transected with fine scissors, and perfusion with cold saline will be initiated at a flow rate of 60 ml/min, clamping the aorta below the diaphragm, but above the origin of the renal arteries. Once perfusion is completed, samples of the kidney and liver will be collected and snap‐frozen in liquid nitrogen. Separate samples will also be immersion‐fixed in 10% non‐buffered formalin overnight at 4°C for histological analyses.

### 2.4 Primary outcome measure and sample size

#### 2.4.1 Urinary albumin excretion

The study is designed to focus on testing differences in urinary albumin excretion between dark phase TRF treated group (Group B) relative to the equicaloric light and dark‐phase intake group (Group C), both being comparable for reference to a positive ad libitum‐fed control group (Group A). Urinary albumin concentration will be measured using an electrochemiluminescence‐based rat kidney injury marker ELISA kit (K15162C Meso Scale Discovery, Rockville, MD, USA) and expressed as both the albumin excretion rate in microgram per hour and albumin–creatinine ratio (ACR) in units of microgram per milligram. Urinary creatinine will be assessed by colorimetric Jaffe reaction assay (500701 Cayman Chemical, Ann Arbor, MI, USA). The kit will also allow for the determination of urinary excretion of neutrophil gelatinase associated lipocalin, kidney injury molecule‐1 and osteopontin as secondary end‐points.

#### 2.4.2 Sample size

Power analysis for one‐way ANOVA has been used for sample size calculations. Calculations are replicated in G*Power https://www.psychologie.hhu.de/arbeitsgruppen/allgemeine‐psychologie‐und‐arbeitspsychologie/gpowergenerated and In Vivo Stat https://invivostat.co.uk/wp‐content/user‐guides‐v4/Power_analysis‐one‐way_ANOVA.pdf. Estimates for effect sizes and variance are drawn from the report of Khan and Imig (2011) in which the same model (HFD‐fed adult male SHR rats) was used to study the effect of two angiotensin II type 2 receptor blocker drugs on Met‐S‐associated urinary albumin excretion (mg/day). After 10 weeks of HFD and treatment with drug or vehicle, they observed a significant reduction in urinary albumin excretion from 85 mg/day (SD 19.2, SEM 8) in the vehicle group to 45 mg/day (SD 7.2, SEM 3) in the valsartan group and to 23 mg/day (SD 12, SEM 5) in the telmisartan group (Khan & Imig, 2011).

Based on the above data, the present study is powered to be able to detect a biologically meaningful effect of at least a 40% reduction in mean urinary albumin excretion in the TRF intervention (Group B treatment) versus untreated ad libitum‐fed group. We estimate that the magnitude of the effect size obtained with TRF will be double that of equicaloric light/dark feeding (Group C) because of the propensity of the former to better address risk factors. The analyses of other dynamic secondary endpoints in the study will allow for interrogation and corroboration of this hypothesis.

Using these effect size estimates (Cohen's *d* of 0.92) and the absolute data on albuminuria from the above paper, power analysis was conducted for type 1 and type 2 error rates of 5 and 20%, respectively. The pooled standard deviation was estimated as 1.5‐fold higher than that reported by Khan and Imig (2011), equating to coefficient of variation estimates between 17 and 29%. This results in a statistical power of 81% for a three group comparison with *n* = 6 per group. The minimum number of experimental units per group estimated to be required to retain 80% power is *n* = 5. Therefore, based on effect size estimates, there is scope for attrition on primary outcome measures in one animal per group.

### 2.5 Secondary outcome measures

Other parameters assessed as secondary outcomes are food intake (g/day), body weight (g), blood pressure (mmHg), glucose tolerance test (post‐challenge 2‐h plasma glucose (mmol/l), area under the curve), dark phase urinary C‐peptide/creatinine ratio (a proxy for insulin resistance assessed in 8 h urine collections using an ultrasensitive rat C‐peptide ELISA (90055, Crystal Chem). Using necropsy samples obtained at the end of the study, a semi‐quantitative histological assessment of hepatic steatosis (Liang et al., 2014), quantitative morphometry of renal glomerular volume according to the Weibel and Gomez formula (Alizadeh et al., 2018; Lane et al., 1992; Thomas et al., 2011; Wu et al., 2022, 2021), and semi‐quantitative assessment of glomerulosclerosis with periodic acid–Schiff staining will be carried out. Quantitative gene expression analysis of inflammation markers (monocyte chemotactic protein‐1 and CD68 mRNAs) and markers of fibrotic activation (transforming growth factor β 1 and fibronectin mRNAs) in the kidney will also be assessed.

### 2.6 Planned statistical analyses (Table 1)

Initial data summarisation and cleaning will be completed using GraphPad Prism 9 (GraphPad Software Inc., San Diego, CA, USA). Only rats that complete the study will be included in the statistical analysis. Group distribution will be assessed with the Shapiro–Wilk test. Outlier data points will be screened for initially using the Rout's method; this has been developed to apply if one is unsure of whether there are none, one or more outliers. Detection of a single outlier will be further confirmed by Grubbs's method. Further inferential statistical analysis will be performed using the R package rstatix in RStudio (R version 4.1.1). *P* ≤ 0.05 will be considered statistically significant. Cross‐sectional comparisons of the primary outcome measures of albuminuria (rate and ACR), and the secondary outcomes of glucose tolerance, gene and histopathology will be assessed by one‐way independent group ANOVA with Holm–Šidák's multiple comparisons test with a normal distribution of the data, and by the Kruskal–Wallis test with non‐normal distributions. Serially assessed secondary outcomes (weight, albuminuria, blood pressure, urinary C peptide and renal injury biomarker excretion) that include time and treatment as independent variables will be assessed by two‐way repeated measures ANOVA with the Bonferroni multiple comparisons test with a normal distribution of the data. Non‐normally distributed serial data will be log10‐transformed and assessed by two‐way repeated measures ANOVA. Statistical significance value will be established at *P* ≤ 0.05. Study endpoints will be plotted as boxplots or violin plots using the R package ggplot2 showing individual data points for each animal.

**TABLE 1:** Summary of outcomes and planned statistical analyses.

Outcome		Planned statistical analyses
Primary outcome	Albuminuria: Urinary albumin excretion rate (μg/h) Urinary albumin–creatinine ratio (μg/mg)	One‐way ANOVA or non‐parametric test
Secondary outcomes	Food intake (g/day)	Two‐way ANOVA
	Body weight (g)	Two‐way ANOVA
	Blood pressure (systolic, diastolic and mean arterial pressure) (mmHg)	Two‐way ANOVA
	Urinary C‐peptide excretion: Urinary C‐peptide creatinine ratio (pg/mg)	Two‐way ANOVA
	Glucose tolerance (area under the curve of plasma glucose (mmol/l))	One‐way ANOVA or non‐parametric test
	Urinary tubular biomarkers excretion: Neutrophil gelatinase‐associated lipocalin (NGAL) creatinine ratio (ng/mg) Kidney injury molecule‐1 (KIM‐1) creatinine ratio (ng/mg) Osteopontin (OPN) creatinine ratio (ng/mg)	Two‐way ANOVA
	Histopathology: Glomerular volume (μm^3^) Hepatic steatosis (categorical grading of macrovesicular and microvesicular steatosis) Glomerulosclerosis (digital pathology quantification of Periodic acid–Schiff (PAS) staining (arbitrary units))	One‐way ANOVA or non‐parametric test
	Renal gene expression (mRNA) analyses (arbitrary units): Transforming growth factor β 1 (TGFβ‐1) Fibronectin Monocyte chemotactic protein 1 (MCP1) CD68	One‐way ANOVA or non‐parametric test

#### 2.6.1 Measures to prevent bias

Animals will be randomized between groups to prevent allocation bias (as per section 2.1.2., ‘Stratified allocation’). To avoid analytical bias, all samples retained for analysis will be recorded using a random number generator (https://www.graphpad.com/quickcalcs/randomN2/). This will be carried out by a third party who will keep the random number key and not disclose or share it with the researchers conducting primary sample analyses. Decoding and reassembly of data by the experimental group will be carried out by a third party who will also recode and conceal group identity (A, B or C) by deriving an alternative codename for each.

## AUTHOR CONTRIBUTIONS

All authors have contributed to the conception and design of the study and drafting of the registered protocol. All authors have read and approved the final version of this manuscript and agree to be accountable for all aspects of the work in ensuring that questions related to the accuracy or integrity of any part of the work are appropriately investigated and resolved. All persons designated as authors qualify for authorship, and all those who qualify for authorship are listed.

## CONFLICT OF INTEREST

The authors declare no conflicts of interest.
